# The Overriding of Computerized Physician Order Entry (CPOE) Drug Safety Alerts Fired by the Clinical Decision Support (CDS) Tool: Evaluation of Appropriate Responses and Alert Fatigue Solutions

**DOI:** 10.7759/cureus.31542

**Published:** 2022-11-15

**Authors:** Anjum Naeem, Ali F Alwadie, Abdullah M Alshehri, Lama M Alharbi, Muhammad U Nawaz, Rawad A AlHadidi, Raed S Alshammari, Muath A Alsufyani, Lamis O Babsail, Shahad A Alshamrani, Ayed A Alkatheeri, Norah F Alshehri, Abdullah M Alzahrani, Yahya A Alzahrani

**Affiliations:** 1 Pharmaceutical Care Department, Ministry of National Guard - Health Affairs, Jeddah, SAU; 2 College of Pharmacy, University of Tabuk, Tabuk, SAU; 3 College of Pharmacy, Umm Al-Qura University, Makkah, SAU; 4 Drug Information Center, Pharmacy Department, East Jeddah Hospital, Ministry of Health, Jeddah, SAU; 5 Clinical Pharmacy Unit, Pharmacy Department, East Jeddah Hospital, Ministry of Health, Jeddah, SAU

**Keywords:** cpoe, cds, responses, alert fatigue, alert overriding

## Abstract

Introduction

Most computerized physician order entry (CPOE) software come with clinical decision-support components (CDS) that provide prescribers assistance and notify them about adverse drug reactions. An excessive number of alerts in a repeated and non-relevant manner leads to alert fatigue and enforces physicians and pharmacists to alert overrides. King Abdulaziz Medical City (KAMC) in Jeddah still reports a higher percentage of drug alerts overridden by clinicians and pharmacists. Thus, this study was conducted to evaluate CDS alerts overriding and to determine which alerts are clinically irrelevant and need modifications.

Methods

The study was carried out in the inpatient setting at KAMC in Jeddah, from September 1, 2020, to December 31, 2020. It was designed as a retrospective chart review study that included all red alerts that required comments and were overridden by a physician and pharmacist.

Results

Among 11350 red alerts, potential drug-drug interaction (pDDI), dose, and allergy alerts represent 57%, 41%, and 2%, respectively, of the total alerts. The most common drug-drug interactions (DDIs) in category X were proton pump inhibitors and clopidogrel (9.9%). The appropriate response by prescribers and pharmacists toward allergy alerts was associated with the highest odds compared with the other alerts (p < 0.05) while there is a significant decrease in the odds of appropriate action being taken by both prescribers and pharmacists in the dose screen alerts (p < 0.05). Among all clinical specialties, there is an increased odds of appropriate action being taken by residents and fellows for allergy and dose alerts, respectively, compared to other groups (p < 0.05). For diminishing the unnecessary alerts, we provided 14 alert refinements strategies and advised turning off four alerts. Applying this will terminate 32% of irrelevant alerts.

Conclusion

Our study's findings indicated that a substantial number of alerts are ignored, and the rate of appropriateness varies significantly by alert type and prescriber level.

## Introduction

A computerized physician order entry (CPOE) avoids medication order mistakes caused by ambiguity in handwriting and inconsistency in transcribing. Most CPOE software comes with clinical decision support components (CDS) that play an important role in reducing errors and enhancing patient safety. CDS is defined as "an artificial intelligence tool that was designed to be direct aid to clinical decision making, in which the characteristics of an individual patient are matched to a computerized clinical knowledge base resulting in patient-specific assessments or recommendations" [1,2]. CDS provides prescriber assistance and notifies them about adverse drug reactions (ADR), including drug-related (drug-drug interactions (DDIs), dosing errors, duplicate therapy, drug-herb interaction, drug-food interactions), and allergies [3-5].

Several studies indicated that around 28% of ADRs could be prevented, and this brought important consideration to the use of CDS to minimize patient harm. Have since, many reports arose and confirmed that medication errors, "which occur in 4%-6% of orders," can be limited by activating CPOE and CDS. Despite the fact that improving patient safety is a primary reason for adopting CDS, there are also financial implications, as CDS has resulted in substantial cost savings [6]. According to a survey, physicians on duty regularly issued about 56 warnings and spent 49 minutes treating them, making alerts an important part of the daily care workflow [6].

Notwithstanding the success of the initial CDS reports, the assessment of the CDS tool has not consistently shown improved patient outcomes. The non-compliance behavior of many clinicians toward alerts 'alert overrides' appears in 49%-96% of received alerts and is a possible obstacle to such success [6,7]. Thus, the CDS tool will be ineffective if alerts are not properly handled or if clinicians do not change their behavioral responses to an acceptable and relevant alarm.

An excessive number of alerts in a repeated and non-relevant manner leads to alert fatigue and enforces physicians and pharmacists to alert overrides [6-9]. Several studies considered the high rate of the overridden alert as an acceptable and justifiable action due to the irrelevance of the alert, the patient's tolerability of the drug, and the clinician's aim to monitor the patient. Additionally, statistical modeling has been used to evaluate the manageable determinants of alert overrides. Numerous factors influence the overridden process, including personal factors (e.g., prioritization, workflow load), patient and clinician features, triggering substance, alert rate, and response type required. On the other hand, some alerts pop up inappropriately, and adhering to alert warnings could cause a patient harm. Thoughtful evaluation of the suitability of the alerts is essential to distinguish such undesirable, unintended consequences [6]. Numerous attempts have been made to reduce override rates in addition to improving CDS alerts. Currently, there is no universally applicable mechanism for avoiding false positive warnings (which distract clinician time and attention) and false negative alerts (which quietly put patients at risk) [10,11].

King Abdulaziz Medical City (KAMC) in Jeddah still reports a higher percentage of CDS alerts overrode by both clinicians and pharmacists, some of them may be associated with adverse drug reactions and, therefore, patient safety is jeopardized. Beyond the urgent need to evaluate the overriding CDS alert in this center, the improvement of the appropriateness of the CDS alert should also be considered. To the best of our knowledge, no study has been carried out to evaluate the CDS alert overriding by KAMC staff. The current work was conducted to evaluate the CDS alert overriding and determine which alerts are clinically irrelevant and need modifications.

## Materials and methods

Place of study

The study was carried out in the inpatient setting at KAMC in Jeddah, from September 1, 2020, to December 31, 2020. It was designed as a retrospective chart review study that included all red alerts that required comments that were overridden by a physician and pharmacist and occurred on an inpatient related to DDI, allergy, and dose screening. All other types of alerts (orange and yellow), outpatient overridden alerts, and alerts that occurred outside the time of the study periods were excluded. The current work was approved by the Ethics Committee of the King Abdullah International Medical Research Center (KAIMRC) number (NRJ21J/105/04).

Determine the appropriateness of the provider's action

**Figure 1:**
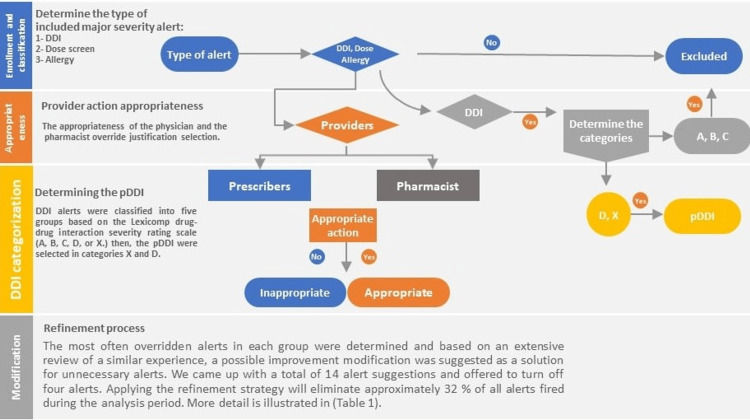
Flow chart for red alert selection and modification

All override response documentation from providers (physician and pharmacist) was analyzed based on the appropriateness criteria for each type of red alert type (e.g., DDI, drug allergy, and drug dose). BESTCare® 2.0A equips the CDS tool to offer suggestions for medication-related problems. These warnings' sense was sourced from Medi-Span (Master Drug Database (MDDB), Wolters Kluwer Health, Inc.). Of the seven alert categories provided by BESTCare 2.0A, three were included in this study: drug-drug interactions, drug-dose screening, and drug allergy alerts. Four degrees of severity have been impeded; level 1 indicates the most significant severe alarms with an obligate rationale for the override with a red color code to make it easier to distinguish between the four levels (level 2 - level 4 not included). BESTCare provides a non-customized reasons list for all categories of red alarms. Before overriding a level 1 warning, providers (physician and pharmacist) must select a justification from a drop-down list that includes 'benefit outweighs risk,' 'dose checked and confirmed,' 'allergy not proven,' 'not clinically significant,' 'compatibility confirmed,' 'dose altered for patient characteristics,' 'the patient is being monitored,' and 'side effect, not allergy.' To determine DDI alerts, we assessed the frequency of DDI alerts during the research period and chose to focus on significant DDIs. We classified the DDI alerts into five groups based on the Lexicomp drug-drug interaction severity rating scale (A, B, C, D, or X) and then selected the potential DDI (pDDI) pairs in categories X and D as shown in Figure 1.

Data collection

The total number of all red alerts during the study period was identified through the BestCare® coordinator. All the data for the red alerts were collected and saved using Microsoft Excel 2010 (Microsoft Corporation, Redmond, WA) and protected by a password key to ensure data confidentiality. The data collecting sheet included the following data: drug name, advisory comment, alert type (DDI, dose screen, and allergy), prescriber level (resident, fellow, and consultant), prescriber comment, pharmacist comment, appropriateness of prescriber action, appropriateness of pharmacist action, DDI category (A, B, C, D, and X). The appropriateness of each alert was determined based on predetermined criteria. Moreover, the pDDI was determined by "using the Lexicomp drug-drug interaction severity rating scale," selecting pDDI in categories X and D.

Primary endpoints

- To determine alerts that are consistently being commonly overridden and their association with an appropriate action taken by either prescribers or pharmacists.

- To assess the distribution of appropriate responses for red alerts among different prescribers’ levels (pDDI, overdose, and allergy).

Secondary endpoints

- To decrease the number of unnecessary red alerts that pop up to the pharmacist during the verification process.

Statistical analysis

All statistical analyses were performed by using the statistical package for the social sciences (SPSS) version 26.0 (IBM Corp., Armonk, NY). The association of appropriate responses toward red alerts overridden by pharmacists and different prescribers' levels was performed by using the chi-square test. All reported p-values were two-sided, and a p-value of <0.05 was considered statistically significant.

## Results

**Figure 2:**
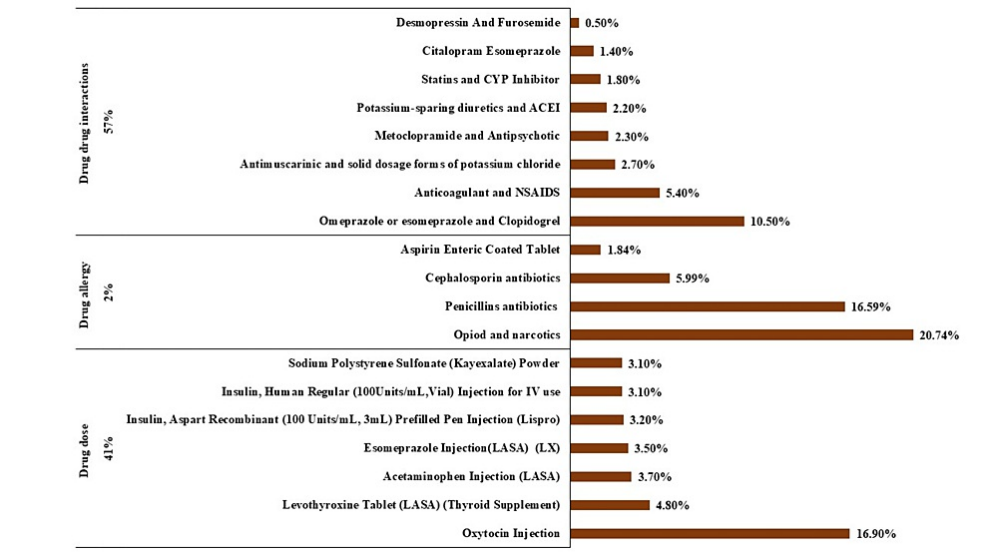
Distribution of red alerts that are consistently being overridden

Approximately 176612 orders were prescribed by physicians and verified by pharmacists during the study period. A total of 11350 red alarms were fired from the CPOE system; making 6.4 alerts/100 orders. The provider response rate was 100% since the system required them to take action for all red alerts. There were 6498 DDIs (57%), 4707 dose-screen alerts (41%), and 217 drug allergy alerts (2%) of all fired alarms. Among 6498 DDIs identified by the CDS tool, the most common DDIs in category X were PPI and clopidogrel (680, 9.9%), anticoagulants, and non-steroidal anti-inflammatory drugs (NSAIDs) (350, 5.4%), antimuscarinic and solid dosage forms of potassium chloride (175, 2.3%), and metoclopramide and antipsychotic (150, 2.3%). Figure 2 shows red alerts that are commonly overridden.

Red alerts were associated with an appropriate action taken by either prescriber or pharmacist

There is an increased odds of appropriate response among the prescriber toward the pDDI alerts (OR = 1.32, CI 95% =1.205 - 1.458, p < 0.05) compared to the other alerts. While there is a significant decrease in the odds of appropriate action taken by the prescriber in the dose screen red alerts (OR = 0.7, CI 95% = 0.6626 - 1.204, p < 0.05) compared to the other alerts. In addition, the appropriate response by prescribers for allergy alerts was associated with the highest odds compared with the other alerts (OR = 1.65, 95% CI = 1.096 - 2.505, p < 0.05). Furthermore, there was a significant decrease in the appropriate response among pharmacists in the dose screen alerts compared to the other alerts (OR = 0.73, 95% CI = 0.67 - 1.21, p < 0.05). While our results demonstrated a significant increase in the appropriate action taken by pharmacists toward the allergy alerts (OR = 1.54, 95% CI = 1.07 - 2.23, p < 0.05) as compared to other alerts.

The distribution of appropriate responses for pDDI, overdose, and allergy alerts among different levels of prescribers

**Figure 3:**
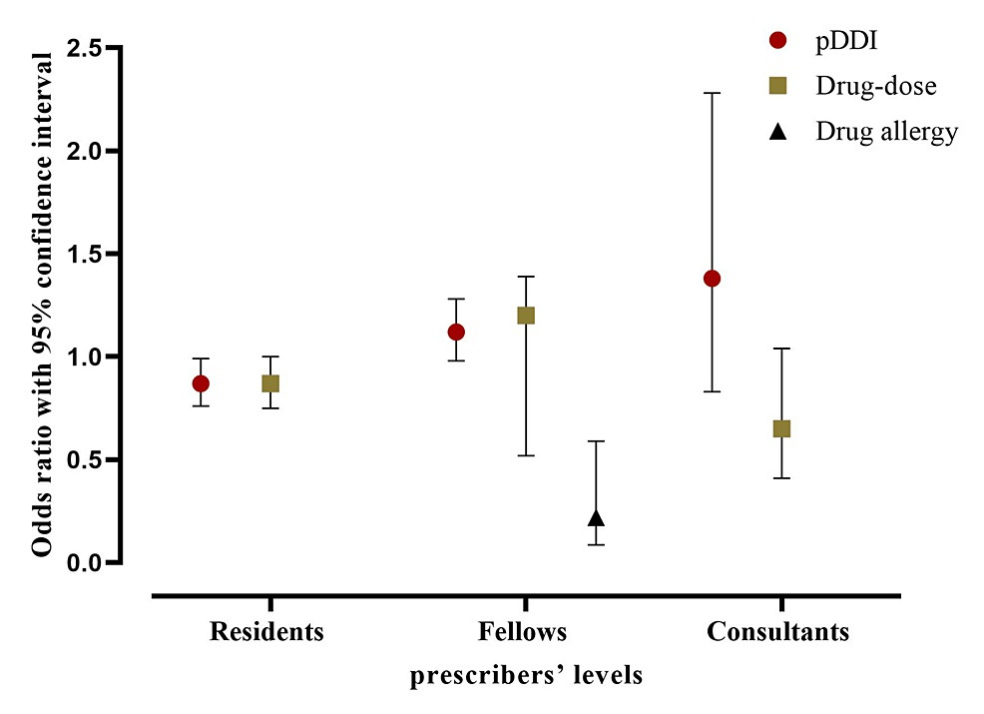
The odds ratio and the 95% confidence interval (CI) of the appropriateness of action taken among different prescribers’ levels

Among all clinical specialties, residents accounted for most of the alerts (6168, 54%), with 4998 (44%) of those alerts being overridden appropriately. The fellow experienced around 5018 red alerts and 37% of them were handled properly. The odds of appropriate response were significantly decreased among residents toward the pDDI alerts compared with those handled properly by fellows and consultants (OR = 0.87, CI 95% = 0.76 - 0.99, p < 0.05). While there is an increased odds of appropriate action being taken by residents for drug-allergy alerts (OR = 5.31, CI 95% =1.79 - 14.68, p < 0.05) compared to other groups. Moreover, the odds of appropriate response were significantly higher among fellow groups toward the drug-dose screen alerts (OR = 1.20, CI 95% = 0.52 - 1.39, p < 0.05) in comparison to other groups. Although the appropriate action taken by fellows in drug allergy alerts was significantly associated with lower odds compared with other prescribers’ levels (OR = 0.22, CI 95% = 0.09 - 0.59, p < 0.05). In addition, the results showed a non-significant increase in odds of appropriate consultant action for pDDI, drug-dose screen, and drug-allergy override alerts compared to the other groups (p>0.05). Figure 3 showed the odds and 95% CI for the seven comparisons related to the appropriateness of action taken by the different prescribers’ levels.

Diminishing unnecessary red alerts firing to the pharmacist during the verification process

To find alerts that could be improved, we looked at the most often overridden alerts and decided to focus on pDDIs, the dose screen listed in the Appendix. We came up with a total of 14 alert suggestions, and we suggested turning off 4 alerts. Applying the refinements strategy will eliminate approximately 32% of all alerts fired during the analysis period (Appendix).

## Discussion

This study found that the appropriate responses of prescribers to allergy and pDDI alerts were 1.6 and 1.3 times higher than dose screen alerts (p<0.05) while pharmacists' appropriate action toward the allergy was 54% higher than the other alerts. Our findings are slightly different from those of the existing literature that reported a higher proportion of appropriate overrides toward dose red alerts. Rehr et al. reported that 21 of 30 (70%) dose alert overrides were appropriate [12]. The possible explanation behind the lower appropriate response to dose alerts in our results is that several variables should be considered during prescribing particular medications, including the patient's weight, renal function, age, liver function status, and concurrent medication usage in the development of CDS alerts for patients. Excluding these factors in our CDS tool, in addition to the irrelevant information that the alerts were built on and used in the wrong situation, makes a significantly higher percentage of dose screen alerts that are not clinically relevant or have very little clinical value, thus leading to desensitization of the providers, causing them to overrule both legitimate and clinically irrelevant alarms. All of these technical issues required substantial modification [12,13].

In the current work, drug allergy alerts had the most appropriate responses from both prescribers and pharmacists compared to other alert classes (OR = 1.65, OR = 1.54, respectively; p < 0.05). These appropriate responses do not necessarily cover the most critical drawback of the CDS tool in firing the allergy alerts, which is the inability of CDS to distinguish between allergies, sensitivities (intolerances), and adverse drug reactions, all of which were handled equally by our CDS tool. Thus, it is likely that most firing alerts for medication allergies were erroneous [14]. More clearly, our interpretation of several allergy alerts showed that itching and rashes were the most prevalent symptoms observed in patients with "allergies" to certain opioids, as indicated in their allergy profile. It is important to note that these symptoms are not true allergic reactions, but just apparent adverse effects of or sensitivities to opiates' pharmacologic activity [15,16]. Therefore, rather than continually alarming about probable "allergies" to ordered narcotics, we would advocate only creating an alert when the requested drug is an exact match to the drug on the allergy list. Additionally, improved methods for differentiating actual allergies from sensitivities should be studied to document "allergy lists" in CPOE. Another concern was noticed that the CDS tool did not recognize cross-sensitivity correctly in some cases. An example of this is piperacillin-tazobactam with normal saline and vancomycin with normal saline. The CDS tool was designed to consider normal saline as part of the allergenic drug and to trigger unnecessary alerts when vancomycin is used concomitantly. This example demonstrates the importance of alert design (content and physical features) when developing alerting systems; system modification based on a comprehensive understanding of these elements has been found to increase alert acceptance rates [17].

Our findings indicated that 57% of all alerts were related to pDDI. These findings are in agreement with previous studies that found DDIs had the highest override rate (87%) and generate a large number of useless alerts due to a lack of relevance and specificity [18-21]. As a result, it is unsurprising that hospitals implementing alert optimization methodologies prioritized boosting the relevance of alerts to the local environment [22]. The appropriateness of the override is more crucial to patient care than the number of alert overrides. Inappropriate overrides have been shown to increase the likelihood of possible and definitive unfavorable patient outcomes by a factor of six [23]. To minimize unnecessary warnings and provide clinically useful alerts, a DDI knowledge base must carefully assess the source of severity information. Although most CDS systems provide evidence for their warnings, physicians should be aware that some rely on theoretical interactions involving known inhibitors, inducers, and substrates of the CYP enzyme system. Many of these DDIs lack clinically relevant reports to support their claims. One of the most notable examples of these DDIs that accounted for 10.5% of the total pDDI alarms in our work is proton pump inhibitors (PPIs), which are frequently administered in conjunction with clopidogrel [24]. Multiple investigations have been done to investigate the clinical implications of this interaction's possible lower antiplatelet efficacy of clopidogrel. According to two retrospective investigations, PPIs were either related to increased cardiac adverse events in acute coronary syndrome patients or decreased cardiac adverse events in PPI non-users [25,26]. On the other hand, several studies have also found that the frequency of cardiovascular adverse events does not vary when omeprazole is administered concurrently with clopidogrel [27-29]. In addition, PPIs including rabeprazole, lansoprazole, dexlansoprazole, and pantoprazole do not show mechanism-based inhibition in in-vitro investigations using human liver microsomes. Lansoprazole and pantoprazole have less powerful antiplatelet effects than omeprazole [30-32]. As a result of the findings presented here, switching to a PPI other than omeprazole or esomeprazole and downgrading this level of alerts that lacks very well-designed clinical trials to conclude such potential drug interactions that jeopardize patient life might be beneficial. Along the same lines, monitoring DDIs involving QTc prolongation (QT-DDIs) is another tricky issue. Clinical data, such as patient characteristics and laboratory findings, can be included in the more advanced CDS tool to help manage the risk of QT-DDIs, increase the specificity of alarms, and reduce the alert rate. Furthermore, this advanced CDS tool should be able to distinguish between patients at low and high risk of experiencing QTc prolongation. The accuracy of QT-DDI alerts and QT-DDI risk management can both benefit from an individual patient's risk profile [33]. Hyperkalemia is another critical problem related to DDI, Uijtendaal et al. observed that DDI-induced hyperkalemia occurred in 10% of hospitalized patients who received at least one potassium-increasing drug [34]. In the approach, the development of context-specific alarms for potassium-increasing DDIs and patient-specific risk assessments for hyperkalemia will help reduce the number of unnecessary alerts. This finding broadly supports the work in this area by KM Muylle et al. (2020) who found that the optimized CDS, which uses context factors for the individual risk assessment of hyperkalemia, significantly reduced the alert burden by 92.8% [35].

## Conclusions

In this retrospective study, we described the red alert override patterns by providers and determined which alerts are clinically irrelevant and need modifications. We found that more than half of the alerts were pDDI, and the drug allergy alerts had the most appropriate responses by both prescribers and pharmacists when compared to other alert classes (OR = 1.65, OR = 1.54, respectively; p < 0.05). For diminishing the unnecessary alerts, we provided 14 alert refinements strategies and advised turning off four alerts. Applying this will terminate 32% of irrelevant alerts. The present work could aid clinical decision support system (CDSS) implementers by providing knowledge regarding practitioners’ alert overriding behaviors as well as the need for the implementation of an advanced CDSS tool that includes specific information for the patient, DDI members, and patient risk factors. We anticipate that our recommendations can lead to consistent and clinically relevant content for interruptive DDIs, and thus decline alert fatigue and enhance patient safety.

## Appendices

**Table 1:** Selected unnecessary red alerts among different groups recommended being corrected or downgraded A: Downgrade category; B: Minor modification required; C: Major modification required; D: Unnecessary alert (turning off)

Drug	CDS tool Comments	Refinement Group	Alert Refinement	Recommendation
Omeprazole or esomeprazole and clopidogrel	The use of omeprazole or esomeprazole may lead to reduced ability of clopidogrel to inhibit platelet aggregation and increase the risk of subsequent cardiovascular events.	A	Downgrading (scaling down) this DDI alert to level 2	Despite pharmacodynamic studies suggesting the ability of omeprazole to attenuate the antiplatelet effect of clopidogrel. This interaction does not appear to translate into a higher cardiovascular risk, and there is ongoing debate regarding the most effective strategies for addressing this issue.
Anticoagulant and NSAIDS	The risk of bleeding induced by anticoagulant may be increased by coadministration of NSAID	B	Implementation of an advanced CDS tool that includes specific information for the patient, DDI members, and patient risk factors will guarantee the delivery of an integrated alert by CDS.	All pharmacological interactions resulting in 'bleeding' would be linked to applying concepts such as haemoglobin, platelets, and PT/INR. To detect and standardize each interaction's consequences
Citalopram and Esomeprazole	Plasma concentrations and toxic effects of citalopram hydrobromide may be increased by concomitant administration of esomeprazole. Specifically, citalopram doses greater than 20 mg/day are not recommended in patients receiving esomeprazole tablets according to official package labeling due to the risk of QT prolongation.	C	Implementation of an advanced CDS tool that will help in identifying patients who were at increased risk for developing QTc-prolongation	A clinical decision support tool may be a helpful tool for managing QT-DDIs through a structured approach that enables prescribers to get more precise recommendations.
Potassium chloride	All solid dosage forms of potassium chloride are contraindicated for use in any patient receiving anticholinergic agents	B	Alert only activates when an anticholinergic drug with extended-release potassium is prescribed.	The risk of gastric injury caused to anticholinergics' delayed gastric emptying is only for the prolonged potassium dosage forms
Antimuscarinic and solid dosage forms of potassium chloride	Coadministration of antimuscarinic (oxybutynin, hyoscine-n-butyl bromide, and glycopyrrolate) and solid dosage forms of potassium chloride (e.g., klor-con) may increase the risk of potassium-induced gastrointestinal mucosal damage.	B	As above.	As above.
Statins and CYP3A4 inhibitors	Plasma concentrations of statins (i.e. atorvastatin, lovastatin, and simvastatin) may be increased when co-administered with potent CYP3A4 inhibitors. Adverse effects, including myopathy and rhabdomyolysis, may occur. Coadministration may be contraindicated in official package labeling.	C	Implementation of an advanced CDS tool that includes; specific information for the patient, DDI members, and patient risk factors will guarantee the delivery of an integrated alert by CDS.	The CDS will incorporate linkages between drug interactions, clinical effects, and related data factors; the CDS tool will then provide an alert, if necessary, based on this information.
Desmopressin and furosemide	The risk of desmopressin-induced hyponatremia may be increased by coadministration with furosemide.	B	Alert will be fired if the last sodium level was 130 mEq/L or if there hasn't been a sodium level in 7 days.	Serum sodium monitored routinely.
Entresto (sacubitril / valsartan) and potassium-sparing diuretics. Potassium ACEI and/or ARB	The risk of hyperkalemia may be increased when potassium-sparing diuretics are co-administered with angiotensin-II receptor antagonists.	B	Alert only when the previous potassium level was > 5.5 mEq/L or when no potassium level has been obtained in 7 days.	Serum potassium monitored routinely. Risk of unnoticed hyperkalemia present when the patient is not monitored or has high serum potassium at initiation.
Augmentin 1g (875mg amoxicillin + 125 mg clavulanic acid)	Single Dose Message: The single dose of 1 gram exceeds the maximum single dose of 875 mg. The usual daily dose is 1,750 mg.	B	Alert only fires when Augmentin is ordered in a dose > 1 g per dose	The CDS tool designed to identify the augmentin dose based on the amoxicillin alone not on the combination.
Levothyroxine (Thyroid Supplement)	Single Dose Message: The single dose of 125 mcg exceeds the maximum single dose of 100 mcg. The usual daily dose is 12.5 to 100 mcg.	C	Alert only fires when Levothyroxine is ordered in a dose > 300 mcg per day.	The range of required doses is wide and varies from 50 to ≥200 mcg/day.
Insulin, Aspart Recombinant (100 Units/mL, 3mL) Prefilled Pen Injection (Lispro)	Single Dose Message: The single dose of 18 units exceeds the maximum single dose of 16.5 units. The usual daily dose is 2.75 to 132 units.	C	Alert only fire when insulin aspart dose exceeds the maximum dose accompanied by hypoglycemia	The usual maintenance dose is 0.4 to 1 unit/kg/day in divided doses (ADA 2021). The alert should be built based on the updated weight and dose range, rounding to the nearest 10 units. The dose adjustment in some patients required a higher dose than the maximum labeled dose, so clinical decisions also should be considered.
Insulin, Human Regular (100Units/mL, Vial) Injection for IV use	Single Dose Message: The single dose of 10 units exceeds the maximum single dose of 7 units. The usual daily dose is 3.5 to 7 units.	C	Alert only fire when insulin aspart dose exceeds the maximum dose accompanied by hypoglycemia	Usual TDD maintenance range: SUBQ: 0.4 to 1 units/kg/day in divided doses (ADA 2021). The alert should be built based on the updated weight and dose range, rounding to the nearest 10 units. The dose adjustment in some patients required a higher dose than the maximum labeled dose, so clinical decisions also should be considered.
Sodium Polystyrene Sulfonate (Kayexalate) Powder	Single Dose Message: The single dose of 30 grams exceeds the maximum single dose of 15 grams. The usual daily dose is 15 grams.	C	Alert only fires when Sodium Polystyrene Sulfonate is ordered in a dose > 30 grams per dose.	Oral (preferred route): 15 to 30 g once; may repeat up to 4 times per day as needed based on serum potassium levels. Maximum daily dose: 60 g/day.
Methotrexate Injection	Administration of Methotrexate Injection (LASA) [85 mg Intramuscular For 1 Day] is contraindicated in Pregnancy.	D	Suppress	Usually used in ectopic pregnancy
Methylergonovine Maleate Injection	Administration of Methylergonovine Maleate Injection [0.2 mg Intramuscular For 1 Day] is contraindicated in Pregnancy.	D	Suppress	Usually used after delivery to prevent haemorrhage in the life-threatening situation only
Measles, Mumps, and Rubella Virus Vaccine Live Injection	Administration of Measles, Mumps, and Rubella Virus Vaccine Live Injection [0.5 mL Subcutaneous For 1 Day] is contraindicated in Pregnancy.	D	Suppress	Used after delivery for all mother
Misoprostol	Administration of Misoprostol (LASA) [800 mcg Sublingual For 1 Day] is contraindicated in Pregnancy.	D	Suppress	Used in early pregnancy for delivery or abortion
Oxytocin Injection	Daily Dose Message: The daily dose of 20 units exceeds the usual dose of 0.72 to 14.4 units.	C	Alert only fires when the administered dose exceeds 20 milliunits/minute the alert should be adjusted as per our hospital guideline	Used in Induction or stimulation of labor. Oxytocin dose should be adjusted as per our hospital guidelines.
